# Supplementary material for: Phylogenomics picks out the par excellence markers for species phylogeny in the genus Staphylococcus
Source: PeerJ. 2018 Oct 24;6:e5839. doi: 10.7717/peerj.5839 (PMC6203942; doi:10.7717/peerj.5839)
Supplement: Table S4 — GO enrichment analysis via Blast2GO PRO. [file peerj-06-5839-s004.docx]

**Supplementary Table 4.**

List of enriched GOs using the 177 single gene families (SGF).
