# Supplementary figures and images for: Phylogenomics picks out the par excellence markers for species phylogeny in the genus Staphylococcus
Source: PeerJ. 2018 Oct 24;6:e5839. doi: 10.7717/peerj.5839 (PMC6203942; doi:10.7717/peerj.5839)

0.02

rpoB  
Phi test p value:0.0000282

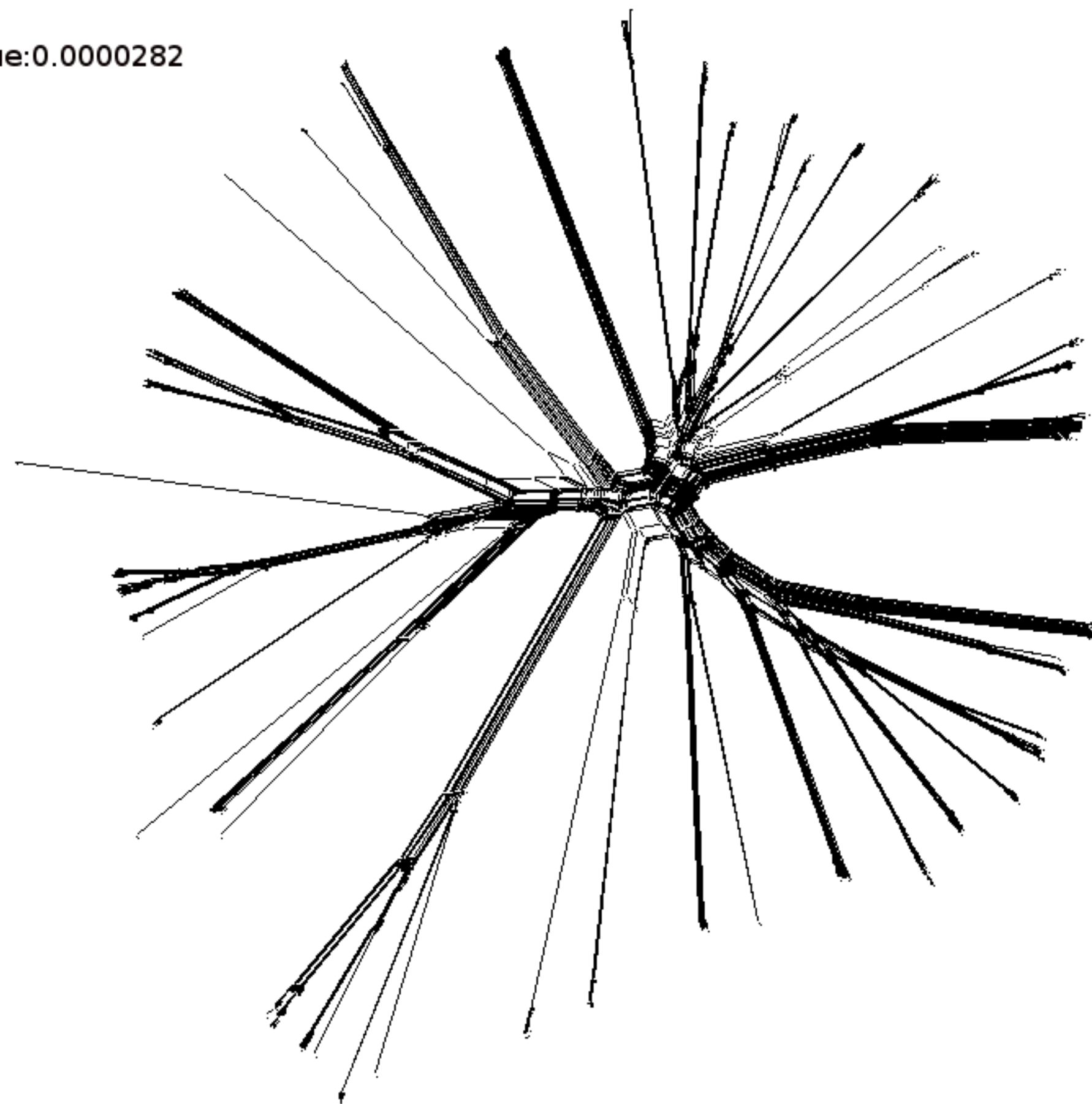

0.02

tuf  
Phi test p value:0.0000804

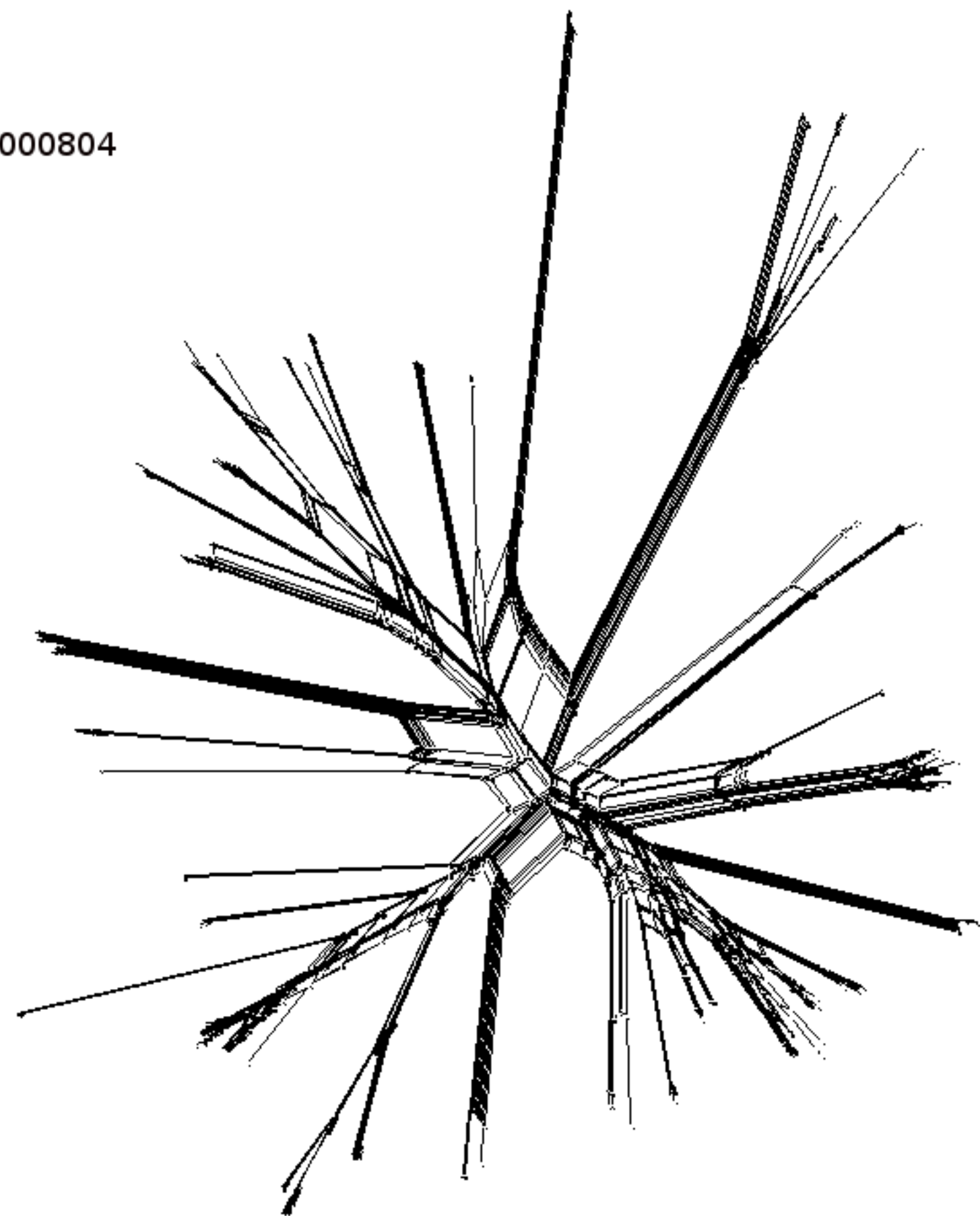

Supplement: Figure S1 — Phylogenetic networks built with SplitsTree, for two (rpoB and tuf) of the usual taxonomic markers. These markers are part of the 208 single copy genes but failed the recombination test. Strain labels are not shown for sake of clarity. [file peerj-06-5839-s005.pdf]

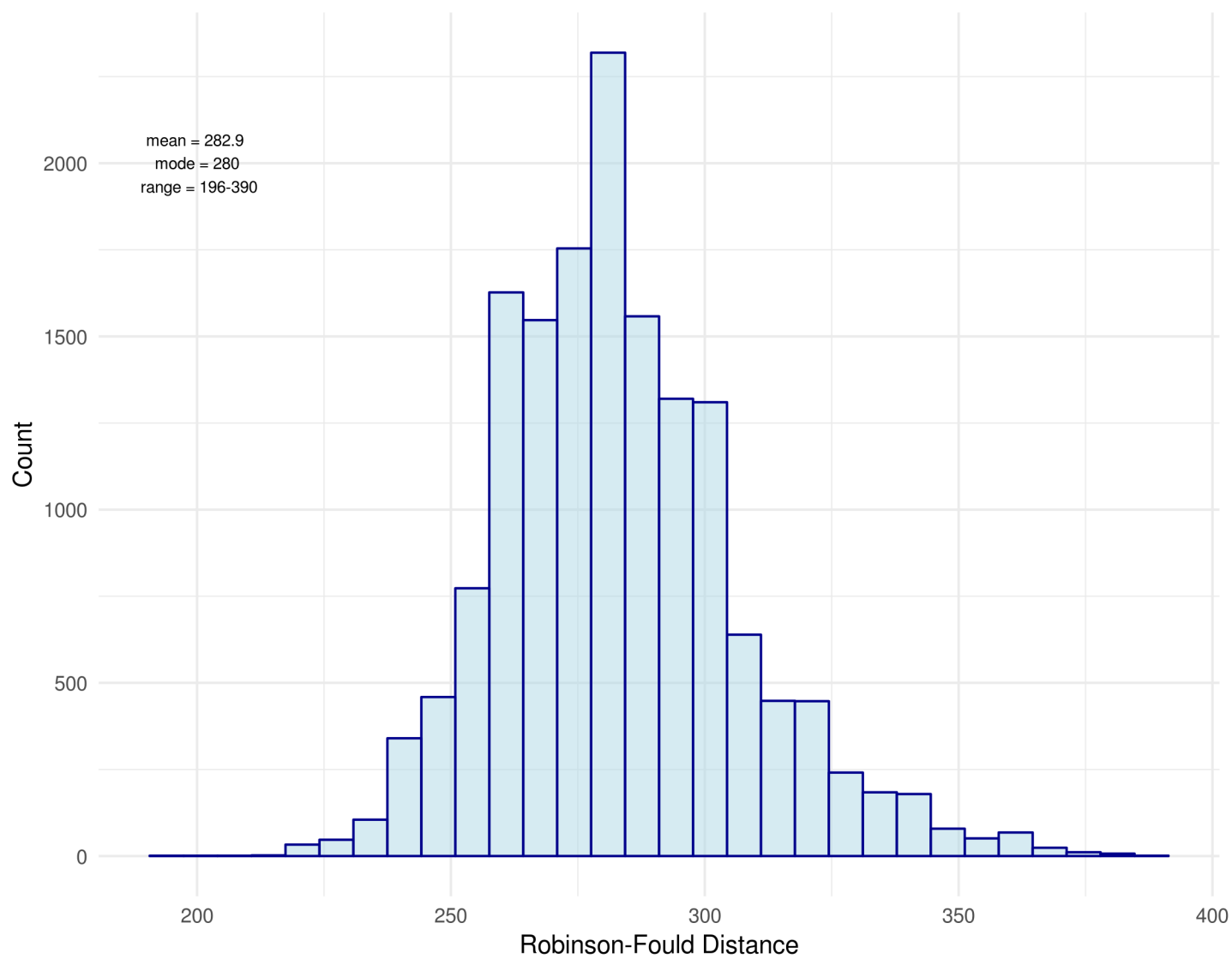

Supplement: Figure S2 — Histogram of the Robinson-Foulds distances estimated between all the SGF trees. There were no two identical topologies and on average topologies are 47% similar to each other. [file peerj-06-5839-s006.pdf]

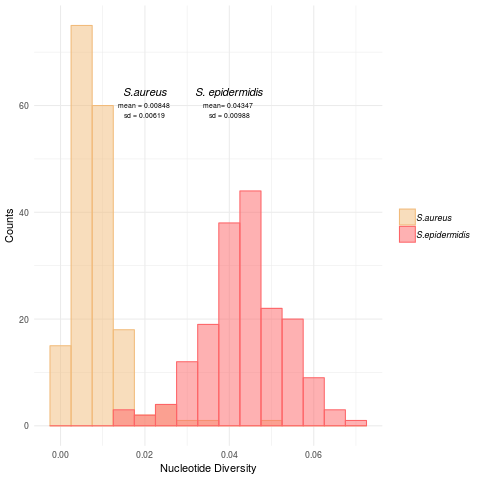

Supplement: Figure S3 — Histograms of the intra-species nucleotide diversity for S. aureus and S. epidermidis. [file peerj-06-5839-s007.png]
